# Cognitive-Behavioural Therapy for Inflammatory Bowel Disease: 24-Month Data from a Randomised Controlled Trial

**DOI:** 10.1007/s12529-016-9580-9

**Published:** 2016-07-18

**Authors:** Antonina Mikocka-Walus, Peter Bampton, David Hetzel, Patrick Hughes, Adrian Esterman, Jane M. Andrews

**Affiliations:** 1 School of Nursing and Midwifery and Sansom Institute for Health Research, University of South Australia, Adelaide, 5001 Australia; 2 Department of Health Sciences, University of York, Heslington, York, YO10 5DD UK; 3 School of Psychology, University of Adelaide, Adelaide, 5005 Australia; 4 School of Medicine, Flinders University, Bedford Park, 5042 Australia; 5 Department of Gastroenterology and Hepatology, Flinders Medical Centre, Bedford Park, 5042 Australia; 6 Department of Gastroenterology and Hepatology, Royal Adelaide Hospital, Adelaide, 5001 Australia; 7 Centre for Nutrition and Gastrointestinal Diseases, School of Medicine, University of Adelaide, Adelaide, 5001 Australia; 8 Australian Institute for Health and Tropical Medicine, James Cook University, Smithfield, 4878 Australia

**Keywords:** Cognitive-behavioural therapy, Disease course, Inflammatory bowel disease

## Abstract

**Purpose:**

There is ongoing controversy on the effectiveness of psychotherapy in inflammatory bowel disease (IBD). In the few small studies, cognitive-behavioural therapy (CBT) has been shown to alleviate symptoms of anxiety or depression. However, there is little research on the impact of CBT on physical outcomes in IBD and no studies on long-term effectiveness of CBT.

**Methods:**

The present two-arm pragmatic randomised controlled trial aimed to establish the impact of CBT on disease course after 24 months of observation. The study compared standard care plus CBT (+CBT) with standard care alone (SC). CBT was delivered over 10 weeks, face-to-face (F2F) or online (cCBT). The data were analysed using linear mixed-effects models.

**Results:**

CBT did not significantly influence disease activity as measured by disease activity indices at 24 months (Crohn’s Disease Activity Index (CDAI), *p* = 0.92; Simple Clinical Colitis Activity Index (SCCAI), *p* = 0.88) or blood parameters (C-reactive protein (CRP), *p* < 0.62; haemoglobin (Hb), *p* = 0.77; platelet, *p* = 0.64; white cell count (WCC), *p* = 0.59) nor did CBT significantly affect mental health, coping or quality of life (all *p* > 0.05).

**Conclusions:**

Therefore, we conclude that CBT does not influence the course of IBD over 24 months. Given the high rate of attrition, particularly in the CBT group, future trials should consider a personalised approach to psychotherapy, perhaps combining online and one-to-one therapist time.

## Introduction

Inflammatory bowel disease (IBD), of which ulcerative colitis (UC), Crohn’s disease (CD) and indeterminate colitis are subtypes, is a chronic relapsing illness of the gastrointestinal tract, affecting 2.2 million people in Europe [1], 1.4 million people in the USA [2], 233,000 in Canada [3] and over 75,000 in Australia [4]. Its course and prognosis are uncertain, and the current standard treatments available for maintaining remission are not tolerated by all patients, are not 100 % protective against relapse even in those who are compliant [5] and place patients at risk of substantial side effects [6]. The search for a cure is ongoing.

While a psychological contribution to IBD’s aetiology is at present uncertain, the illness is associated with significant psychological sequelae. Population-based studies have documented a higher life-time prevalence of depression in IBD compared with the community, with estimated rates of 27 versus 12 % [7]. A recent prospective cohort study (*n* = 2007), using data from the participants included in the Swiss IBD cohort from 2006 till 2015, demonstrated a highly statistically significant association between symptoms of depression and clinical recurrence over time (all IBD, *p* < 0.001; CD, *p* < 0.001; UC, *p* = 0.005) [8]. Other studies link co-morbid mental symptoms to higher hospitalisation rates [9] and lower adherence to treatment [10]. Despite these biopsychosocial interrelations, very few IBD patients with mental disorders or mental symptoms receive psychotherapy—less than 40 % according to a recent Dutch study [11]. Part of the reason for low usage of psychotherapy in this population is poor access to psychologists. For example, a recent national audit in the UK showed that only 12 % of IBD services have access to clinical psychology via a defined referral pathway [12]. However, another significant contribution is a paucity of evidence that psychotherapies are effective for symptoms of mental disorders and any somatic complaints in this population. There have been five systematic reviews of psychological treatment for patients with IBD since 2006, collectively evaluating studies of adults and adolescents [13–17]. Despite this number of reviews, only a handful of studies are available for each of the psychological treatment approaches, with the maximum number of studies evaluated in one review being 21 (*n* = 1745) [13].

von Wietersheim and Kessler [16] reviewed 14 studies and concluded that psychological interventions reduced psychological distress, but there was no clear benefit for disease course. They identified two studies with longitudinal follow-up of at least 24 months. A German study examined psychodynamic therapy over 24 months and concluded it had no effect on disease activity, mental health or quality of life [18]. An American controlled retrospective study conducted in 1964 examined psychoanalytical therapy, with an 8-year observation period, and concluded that this therapy resulted in a better disease course than the control condition [19]. In this study, however, most patients in the treatment group (but not the control group) had co-morbid major psychiatric disorders.

Goodhand et al. [17] evaluated 17 studies and concluded that cognitive-behavioural therapy (CBT) was effective for mood disorders and improved quality of life, while the psychodynamic and psychoeducation approaches were found to have little effect on psychological outcomes. They concurred with von Wietersheim and Kessler [16] in that they found psychotherapies (of any type) to have no impact on clinical symptoms of IBD. However, they noted that most studies included patients in remission or a mixed sample of active/inactive IBD, making it more difficult to detect a positive effect. They identified the same studies with a longitudinal follow-up as von Wietersheim and Kessler.

A Cochrane review [13] assessed 21 controlled studies, 19 of which were included in a meta-analysis to evaluate psychological intervention outcomes. They concluded that psychological interventions (pooled together) had no effect on distress, disease activity or quality of life in the unselected IBD patients, with the exception of psychological interventions in adolescents, which improved mood. The review recommended further studies into psychotherapies for subgroups identified as being in need of psychological support and into types of therapy that may be most useful. While the review was carefully done using well-established guidelines, fundamental issues in the approach to what constitutes a psychotherapeutic intervention make its conclusions problematic. The pooling of therapy types, each associated with differing efficacy for psychological outcomes in other clinical settings and some showing no effect, may have diluted any positive effect in the meta-analysis. In fact, the conclusion that psychological therapy was beneficial for adolescents only may have had less to do with the type of participants and more to do with including just CBT interventions. CBT is very well established as evidence-based treatment for depression [20]. Moreover, half the included studies were psychoeducational interventions, which are not really psychotherapies, and have at best questionable efficacy in the general population. As such, it does not seem appropriate to conclude that psychological treatment is ineffective in IBD, based on those studies. The Cochrane review identified three studies with the longitudinal follow-up of at least 24 months. A German study already identified in previous reviews showed no effect of psychodynamic therapy on any of the outcomes [18]. A small study from Brazil examined social support (unclear of what psychological paradigm) for a period of up to 24 months and demonstrated no impact of the intervention on quality of life (no group difference identified) [21]. Another small trial on hypnotherapy plus peer group sessions followed participants for 5 years and reported a positive effect of the intervention on wellbeing (not defined); however, results were not reported in detail [22].

Two recent systematic reviews addressed shortcomings of the prior reviews [14, 15]. McCombie et al. [15] reassessed all the Cochrane review papers, ultimately evaluating 18 controlled studies of psychological treatment, including eight used in the Cochrane review, three that the Cochrane review had excluded and seven published since. They deliberately excluded psychoeducational studies. However, similarly to Timmer et al. [13], they synthesised findings across the various types of psychological treatment. For almost every outcome, they concluded the findings were mixed, with the exception of agreement of positive outcomes for pain and fatigue. CBT and its variants most commonly contributed to positive outcomes as compared with other psychotherapies, while counselling and psychodynamic therapy more typically yielded negative results. They identified just one study which used a longitudinal follow-up of 2 years (already reported by previous reviews) [18].

Like McCombie et al. [15], Knowles et al. [14] excluded psychoeducational studies and included both controlled and uncontrolled studies of the main types of psychological treatment used for adults with IBD (i.e. stress management, CBT, psychodynamic therapy, hypnotherapy). CBT interventions (*n* = 5) consistently resulted in improved psychological distress, with modest but unsustained changes in gastrointestinal symptoms. Stress management studies (*n* = 5) had mixed outcomes, with one reporting improved anxiety and two reporting reduction in clinical disease indices post-treatment. Two of the four psychodynamic studies found reductions in psychological distress, and one reported decreased healthcare utilisation. There was only limited evidence of improved quality of life for any of the treatments. Knowles et al. have identified three studies with longitudinal follow-up, two already reported by previous reviews [18, 19] and one not previously reported [23]. The small and uncontrolled study by Miller and Whorwell [23] tested gut-focused hypnotherapy following their participants for a mean duration of 5.4 years and concluded that hypnotherapy improved disease activity and quality of life.

Interestingly, the majority of the studies included in the above reviews did not look at clinical levels of anxiety/depression and relied on screening measures rather than an interview, which is a gold standard of psychological/psychiatric diagnosis. Thus, it needs to be acknowledged that until studies start being conducted with populations with significant levels of psychopathology rather than low level emotional distress, we may not be certain whether psychotherapy (of any type) helps IBD patients for their associated anxiety, depression and distress.

To summarise the current evidence for the usefulness of psychotherapy in IBD, there is some support for positive effect of psychological treatments (particularly CBT), on anxiety or depression, but no consistent evidence of improved bowel symptoms. Five studies have examined long-term effectiveness of psychotherapy in IBD, with mixed results [18, 19, 21–23], none of which tested CBT.

The present study was designed to address the gaps in knowledge on the long-term role of psychotherapy, and CBT in particular, on disease activity in IBD. Our hypothesis was that since stress is one of the strongest predictors of disease activity [24], learning CBT skills may ‘inoculate’ patients against stresses of everyday life (of any type but specifically related to IBD itself), which, in turn, may lead to less-frequent IBD flares. We have also specifically addressed the management of IBD-related concerns. We thus conducted a randomised controlled trial to investigate whether adding CBT to standard treatment prolongs remission in IBD in comparison with standard therapy alone. Our first analysis (reported elsewhere [25]) covered the period of 12 months and showed no difference in remission rates between groups, with similar numbers flaring at 12 months. Similar results were found for the secondary outcome measures of quality of life and mental health. However, in a sub-analysis, we examined the effect of the intervention in participants classified as in need of psychological support (young, high baseline IBD activity, recently diagnosed; poor mental health; *n* = 74, 34 CBT and 40 controls) and showed that CBT significantly improved the secondary outcome of mental quality of life (*p* = .034, *d* = .56) at 6 months. The present paper reports on 24-month outcome data for the described intervention, with the aim to establish whether CBT has any effect on IBD remission status after 24 months of observation.

## Methods

### Design

We conducted a randomised controlled trial and the detailed methods as well as the data for the first 12 months of observation have been reported elsewhere [25]. Briefly, the study was a two-arm parallel pragmatic randomised controlled trial (RCT), comparing standard care plus CBT (+CBT) with standard care alone (SC). The trial’s design was pragmatic as those in the experimental group were offered a choice of completing the intervention face-to-face (F2F) or online (cCBT).

### Intervention

F2F CBT group met weekly at a tertiary hospital for 10 weeks for 2-h sessions delivered by a qualified clinical psychologist while cCBT group received sessions with the same content online (self-directed). The psychologist’s training and supervision were provided by the Clinical Psychology Department at the Royal Adelaide Hospital. Treatment adherance was maintained by using the same protocol for both groups within the CBT arm. The programme focused on improving coping with IBD. Sessions are presented in Table 1. They can be accessed elsewhere (http://www.tameyourgut.com/). More detail on the programme is available in our original paper [25]. The study was registered in the Australian New Zealand Trial Registry (ACTRN12609000913279).

**Table 1:** CBT programme’s content

Week number	Theme	Activities
1	Education about IBD and introduction to the programme	Video materials about IBD in general and diet in IBD; goal setting
2	Stress and relaxation	What is stress—its physiology, fight or flight response, symptoms of stress, observe yourself in stressful situations; relaxation training—4 voice recorded sessions of relaxation exercises
3	Automatic thoughts and cognitive distortions	Thinking, feeling and behaviour—introducing CBT basic concepts, observe your thoughts and feelings, identify core beliefs; another 2 recordings to practice relaxation
4	Cognitive restructuring	Emotional wellbeing, appraisal of mood, automatic thought—identify and challenge them
5	Exposure and overcoming avoidance	Avoidance and conditioning—how do we learn to be afraid and how do we overcome conditioning; desensitisation; another 2 recordings of relaxation skill building
6	Coping strategies	What is coping, how do we cope with stress and IBD; worry and sleep; relaxation to help you sleep
7	Assertiveness training	Taking responsibility; introducing assertiveness in communication with family and health professionals; learning to say no
8	Relationships and communication	Social support—quality and quantity; maintaining social networks and interests when dealing with IBD; communication strategies
9	Attention and distraction	Techniques to manage IBD-related pain and discomfort—imagery, focus, distraction
10	Maintaining good mental health	Keeping up momentum—how not to forget what you have learnt; review old goals and plan new ones

### Participants and Sample Size

Participants were recruited from two gastroenterology clinics in Australia and had a clinically established diagnosis of IBD, were in clinical remission or had mild symptoms only for at least 3 months, were aged 18 years or over and had competence to consent. Those with serious mental illness (e.g. psychosis, schizophrenia or any other serious mental disorder requiring one-to-one therapy which could not be offered as part of the trial) or receiving psychotherapy elsewhere were excluded. The power calculation was based on the primary outcome measure, remission at 12-month follow-up for the two main groups (cCBT and SC). With 80 patients per arm, the study had 80 % power at the 0.05 level to detect a 20 % difference in the proportion of patients remaining in remission between the +CBT group (0.80) and SC (0.60) arms.

In the original paper [25], we identified a group of patients in need of additional support who appeared to benefit from the CBT intervention at 6 months, with improved mental quality of life (*p* = .034, *d* = .56). This group was created based on the recommendations from other studies regarding who may benefit from psychotherapy in IBD [13, 26], validated by our own clinical judgement of at risk groups. Participants in need of support were defined as either being recently transitioned from paediatric care (aged 18–20 years), having high baseline IBD activity despite being in IBD remission (the Crohn’s Disease Activity Index (CDAI) >180; the Simple Clinical Colitis Activity Index (SCCAI) >5 while the clinician considered them to be in remission), being diagnosed within last 2 years, having poor coping (a score of 20–25 on either the adaptive or maladaptive coping subscale of Brief COPE) or high anxiety or depression (the Hospital Anxiety and Depression Scale (HADS) score for either anxiety or depression subscale ≥15) [25]. Here, we have included this group in a sub-group analysis.

### Measures

The primary outcome measure for the present longitudinal study was IBD remission at 24 months (established using the CDAI [27] for those with CD or the SCCAI [28] for those with UC). The secondary measures were patient subjective view of IBD activity measured by a question *How well controlled is your IBD?*; disease activity measured on the blood parameters: C-reactive protein (CRP), haemoglobin (Hb), platelet, white cell count (WCC), quality of life as measured on the Short Form 36 Health Status Questionnaire (SF-36) [29, 30] and mental health status (anxiety and depression as measured on the HADS [31] and the State-Trait Anxiety Inventory (STAI) [32], stress measured on the Revised Social Readjustment Rating Scale (RSRRS) [33] and coping as measured on the Brief COPE [34] and the IBD Stages of Change Coping Questionnaire (IBDSCCQ) developed by the investigators based on the work by Carr [35] and the Trans-Theoretical Model of behavioural change [36] at 24 months.

### Analysis

The study applied the intention-to-treat principle. The multivariate analyses were conducted using linear mixed-effects models which allowed for the retention of all subjects in the model including those with missing data. A *p* value of less than .05 was considered statistically significant. Data were analysed using the Stata 14 statistical package.

## Results

**Fig. 1:**
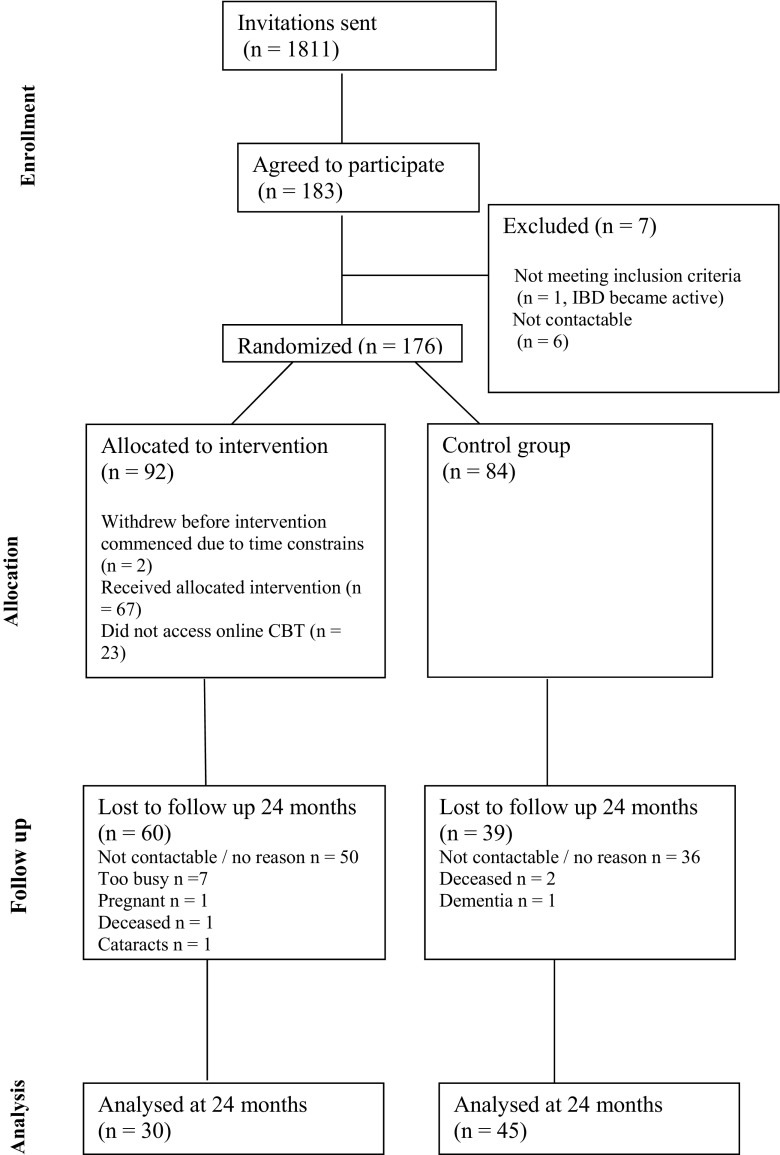
CONSORT diagram showing the flow of participants through the study

The CONSORT diagram (Fig. 1) presents the flow of participants between the study commencement and 24-month data collection. Of the 174 participants who completed baseline measures, 75 remained in the study until the 24-month follow-up (+CBT, *n* = 30; SC, *n* = 45). There was significant attrition observed in both groups, with +CBT group participants dropping from the trial in greater numbers (67 % in +CBT vs. 46 % in SC, *p* = 0.007). The majority of the participants who dropped out of the study were either not contactable (we were allowed by the Research Ethics Committee to attempt contact up to three times) or did not provide the reason for study withdrawal. Of the 174 participants, 68 opted to use cCBT and 22 were allocated to F2F CBT.

### Outcome Measures

Table 2 describes self-reported disease activity at baseline and 24 months. Table 3 reports blood parameters at baseline and 24 months. Table 4 reports self-reported psychological variables at baseline and 24 months, including quality of life, depression, anxiety, coping, stress and stages of change.

**Table 2:** Disease activity at baseline and 24 months

	+CBT				SC			
	Baseline *n* = 90		24 months *n* = 31		Baseline *n* = 84		24 months *n* = 45	
	*n* (%)	Mean (SD)	*n* (%)	Mean (SD)	*n* (%)	Mean (SD)	*n* (%)	Mean (SD)
IBD control								
Very good	27 (30)		11 (36.7)		18 (21.4)		11 (26.2)	
Reasonable	54 (60)		17 (56.7)		56 (66.7)		29 (69)	
Poor	7 (8)		2 (6.7)		6 (7.1)		2 (4.8)	
CDAI (active >150)	15 (17)		2 (6.4)		11 (13.1)		3 (6.6)	
SCCAI (active >3)	8 (9)		6 (19.3)		10 (11.9)		15 (33.3)	
CDAI		110.8 (72.5)		60.4 (91.2)		87.4 (104.8)		53.1 (70.6)
SCCAI		3.4 (1.4)		4.7 (1.7)		3.2 (1.3)		4.9 (2.1)

**Table 3:** Blood results at baseline and 24 months

	+CBT (mean (SD))		SC (mean (SD))	
	Baseline *n* = 69	24 months *n* = 31	Baseline *n* = 70	24 months *n* = 45
CRP	3.6 (5.4)	6.2 (10.2)	6.2 (8.3)	4.4 (10.8)
HB	136.6 (21.8)	140.9 (17.1)	141.9 (15.4)	136.4 (14.7)
Platelet	260.9 (72.8)	258.9 (63.4)	266.4 (63.2)	269.8 (77.1)
WCC	5.9 (1.8)	6.6 (2.4)	6.6 (2.1)	6.4 (1.7)

**Table 4:** Mental health and quality of life at baseline and 24 months

	+CBT (mean (SD))		SC (mean (SD))	
	Baseline *n* = 90	24 months *n* = 31	Baseline *n* = 84	24 months *n* = 45
Physical QoL	46.7 (9.3)	48.8 (10.9)	47 (10.3)	48.8 (8.5)
Mental QoL	44.8 (11.4)	49.8 (8.8)	48.1 (11.5)	48.8 (10.9)
HADS anxiety	7.1 (3.9)	5.3 (4.1)	6.2 (4.3)	5.5 (4.7)
HADS depression	4.3 (3.4)	3.2 (3.7)	4.4 (4.1)	3.9 (3.7)
State anxiety	37.5 (13.1)	32.2 (11.3)	35.9 (13.7)	37.7 (14.7)
Trait anxiety	39.3 (11.9)	33.6 (10.4)	37.4 (11.7)	38 (14.1)
Adaptive coping (range, 20–80 (higher is better))	42.7 (12.8)	43.5 (17.5)	39.5 (11.3)	41.1 (24.1)
Maladaptive coping (range, 8–32 (the lower the better))	10.9 (3.6)	11.6 (10.3)	10.7 (3.7)	12.2 (9.9)
Stress (>300 high stress 150–299 moderate stress <150 low stress)	638.3 (665.9)	338.7 (308.1)	453.6 (490.5)	442.9 (551.1)
TTM stage (range, 2–10 (higher scores mean greater agreement))				
Pre-contemplation	4.5 (1.6)	4.6 (2.2)	4.4 (1.6)	4.9 (2.9)
Contemplation	6.7 (2.1)	6.5 (2.1)	6.3 (2.1)	6.1 (2.7)
Preparation	6.3 (2.3)	6.8 (2.1)	5.7 (2.3)	6.7 (3.1)
Action	5.8 (2.2)	6.4 (2.1)	5.5 (2.1)	6.3 (3.3)
Maintenance	7.1 (1.9)	7.5 (1.6)	7.2 (1.3)	7.6 (2.4)

At the multivariate level (adjusting for baseline), CBT did not significantly change disease activity as measured on the CDAI and the SCCAI at 24 months (CD, *p* = .92; UC, *p* = .87) or blood parameters (CRP, *p* = .61; Hb, *p* = .77; platelet, *p* = .64; WCC, *p* = .59) nor did CBT significantly affect mental health, coping or quality of life (all *p* > .05). Controlling for sex and age did not alter the results. There was a trend towards reduced state (*p* = .29) and trait (*p* = .24) anxiety in +CBT group; however, it was not statistically significant.

At 24 months, of the original group comprising 74 in need of support participants, only 24 remained in the study (6 + CBT and 18 SC). CBT did not significantly change the score on any variable of interest in the participants in need of support (all *p* > .05); however, the experimental and the control groups were numerically imbalanced and the comparisons underpowered.

## Discussion

This longitudinal follow-up to a 10-week CBT intervention for IBD shows no long-term effect of CBT on disease parameters or mental health in the unselected IBD participants. While previous CBT trials have shown positive short-term effect of CBT on mental health in IBD [14], no previous CBT trial has provided follow-up data up to 24 months.

In the present study, the main outcome of interest was disease course. We aimed to test whether gaining CBT skills may ‘inoculate’ patients against stresses of everyday life, which, in turn, may lead to less frequent IBD flares. Psychological stress, reported by large numbers of IBD patients, has been found to be a significant predictor of disease course in IBD in the recent large-scale population-based study [24]. In the present trial, we did not find confirmation of the CBT’s preventive role in reducing IBD’s activity, a finding consistent with the unsustained changes in gastrointestinal symptoms reported by the few previous CBT trials [14]. However, one of the problems in ascertaining the long-term effect of CBT on IBD activity (other than high attrition) is the fact that very few study participants flared during the time of observation, with the large majority of patients reporting no or minimal IBD activity during the 24 months. The exception appears to be some participants with UC. However, we previously documented the problems with the accuracy of the SCCAI in reporting disease activity [25] and given very low numbers of patients reporting poor IBD control on the patient subjective measure, as well as consistent blood test results over time, this worsening of symptoms in UC seems to result from the inaccuracy of the measure rather than being a true reflection of disease activity. A calprotectin test of faecal inflammation could have potentially been used in the present study to ensure that inflammation (and thus disease activity) is reliably detected. Unfortunately at the point of this study’s commencement calprotectin had not been available at the participating sites. In any case, a 24-month follow-up does not appear a sufficient period to capture the course of IBD. Longer time frames of 5 to 10 years may offer more information on the impact of medical and psychological interventions on IBD activity. In addition, future studies could also focus on people with active disease. In the present study, we included only those participants with remitted or mild disease and we have thus likely excluded those individuals in whom emotional distress and disease are most interactive. While examining the long-term disease course in people in remission made sense in testing our hypothesis of whether CBT extends the period of remission, it would be of interest to examine whether psychotherapy performs better in people who are flaring with their IBD than in those in remission.

Retention of participants in the study and ensuring that those assigned to the intervention actually comply with the study requirements (i.e. complete a significant number of sessions) is another obstacle to conducting psychotherapy interventions which rely on online resources. As detailed in our previous paper [25], in the present trial, 25 % of those assigned to the CBT group did not complete a single CBT activity, despite the reminders by the study manager. In the recent CBT trial for IBD which tested a modified version of the present intervention in New Zealand [37], while nearly 90 % of participants in the experimental group accessed the CBT programme, only 26 % downloaded at least half of the resources on offer. This, as well as higher dropout rates in the experimental group versus controls, is in line with other online CBT trials for mental disorders, as evidenced by a recent systematic review [38]. The latter paper points to a significant staff time needed to support depressed clients through online CBT to reduce attrition. Exclusively, online CBT may thus not be an ideal solution for the IBD population, and adding some therapist-run sessions in order to offer a more personalised approach may reduce attrition. Providing standard face-to-face therapy may prove to be the safest option to achieve meaningful therapeutic outcomes in IBD until good quality trials propose new approaches to online psychotherapy delivery. While tailoring therapy to individual needs is more resource intensive up front, it might yield better outcomes over time than the relatively cheap self-directed online psychotherapies. A recent high-quality systematic review and a meta-analysis on depression (not in the gastroenterology context) showed that guided self-help interventions result in effects comparable with face-to-face therapies [39], while unguided self-help produces small, but significant effects [40]. Of closer relevance, a recent systematic review conducted on psychotherapies for irritable bowel syndrome showed that ‘no therapist’ interventions produce little effect while ‘minimal contact’ therapies have the potential to reduce healthcare seeking behaviour and potentially reduce healthcare costs [41]. Interestingly, IBD patients surveyed in a recent New Zealand study (*n* = 102) report preference towards online therapies as compared with face-to-face psychotherapies [42] and thus a search towards an effective online psychotherapy should not cease, yet should clearly involve therapists in the delivery. In addition, in future trials, high attrition rates should be factored into sample size calculations.

Finally, the present trial leans towards the recommendations of the Cochrane review [13] in that psychotherapy does not appear to benefit all IBD patients, and subgroups in need of such an intervention may in fact benefit more than unselected IBD patients. While in our previous paper we showed benefits for the sub-group of participants with identified problems (i.e. the so-called in need of support participants) [25] at 6 months, these trends were not maintained at 24 months. Given a small sample and the fact that the present study had not been powered to show effect in this sub-population, future trials should examine the effectiveness of psychotherapy for groups in need of support over an extended period.

## Limitations

There are relevant psychotherapy factors that were not assessed in this trial such as participant expectancy and for face-to-face modality—connection with therapist, which should be measured in future trials. CBT-specific factors such as, for example, cognitive shifts or reduction in avoidance were not measured and could possibly shed light on why the programme was not effective. Heterogeneity of inclusion criteria, high attrition and low treatment dosage are other limitations relevant to the trial’s findings’ generalisability.

## Conclusions

CBT does not appear to influence disease course in IBD at 24 months in the unselected IBD participants, as measured by disease activity indices and blood parameters, nor does it significantly affect mental health, coping or quality of life. However, a 24-month period of observation was shown not to be adequate to allow for observing changes in disease activity and longer times of observation are recommended for future studies. Given high attrition, particularly in the CBT group, future trials should consider a personalised approach to online psychotherapies, combining online psychotherapy with one-to-one therapist time. The high attrition should also be factored in power calculations. Future trials should also examine the effectiveness of psychotherapy for groups in need of support as opposed to unselected IBD patients.
